# *Lyso*-phosphatidylethanolamine primes the plant immune system and promotes basal resistance against hemibiotrophic pathogens

**DOI:** 10.1186/s12896-020-00661-8

**Published:** 2021-02-03

**Authors:** Ronny Völz, Ju-Young Park, William Harris, Sungkee Hwang, Yong-Hwan Lee

**Affiliations:** 1 Plant Immunity Research Center, Seoul National University, Seoul, 08826 Korea; 2 Department of Agricultural Biotechnology, Seoul National University, Seoul, 08826 Korea; 3 NUTRA-PARK, Seoul, 06732 Korea; 4 Center for Fungal Genetic Resources, Seoul National University, Seoul, 08826 South Korea; 5 Research Institute of Agriculture and Life Sciences, Seoul National University, Seoul, 08826 Korea

**Keywords:** *Lyso*-phosphatidylethanolamine, Plant immunity, Salicylic acid, ROS, *Pseudomonas syringae*

## Abstract

**Background:**

*Lyso*-phosphatidylethanolamine (LPE) is a natural phospholipid that functions in the early stages of plant senescence. Plant innate immunity and early leaf senescence share molecular components. To reveal conserved mechanisms that link-up both processes, we tried to unravel to what extent LPE coordinates defense response and by what mode of action.

**Result:**

We found that LPE-treatment induces signaling and biosynthesis gene expression of the defensive hormone salicylic acid (SA). However, jasmonic acid and ethylene triggered gene induction levels are indistinguishable from the control. In accordance with gene induction for SA, oxidative stress, and reactive oxygen species (ROS) production, we detected raised in-situ hydrogen peroxide levels following LPE-application. Yet, ROS-burst assays of LPE-pretreated plants revealed a reduced release of ROS after PAMP-administration suggesting that LPE interferes with an oxidative burst. Our data refer to a priming effect of LPE on SA/ROS-associated genomic loci that encode pivotal factors in early senescence and considerably improve plant basal immunity. Thus, we challenged *Arabidopsis thaliana* with the hemibiotrophic pathogen *Pseudomonas syringae*. Consistently, we found an increased resistance in the LPE-pretreated *Arabidopsis* plants compared to the mock-pretreated control.

**Conclusions:**

Our results underscore a beneficial effect of LPE on plant innate immunity against hemibiotrophs. Given the resistance-promoting effect of exogenously applied LPE, this bio-agent bears the potential of being applied as a valuable tool for the genetic activation of defense-associated traits.

**Supplementary Information:**

The online version contains supplementary material available at 10.1186/s12896-020-00661-8.

## Background

Phospholipids possess a wide spectrum of biological functions, including the storage of energy, contribution to signaling transduction, and the structural integrity of cell membranes. Notably, phospholipids regulate plant-microbe interactions by stimulating the defense signaling system. Many different phospholipids can be cleaved by specific phospholipases to generate second messengers [1–4]. Phospholipase C and phospholipase D are the key enzymes involved in the generation of phospholipid-derived second messengers, phosphatidic acid, diacylglycerol, and inositol 1,4,5-trisphosphate [5]. Phospholipase A2 hydrolyzes, the structural phospholipid, phosphatidylethanolamine and generates *lyso*-phosphatidylethanolamine (LPE) *in planta* as a minor component of cell membranes [6, 7].

LPE was reported to stimulate mitogen-activated protein (MAP)-kinase signaling cascades [8] and to promote the activation of phenylalanine ammonia-lyase and extracellular acid invertase [9], which are considered to be important metabolic enzymes and pathogenesis-related proteins [10, 11]. In tomatoes, LPE accumulates in wounded leaves and is systemically enriched in non-wounded leaves of injured plants [4]. Nowadays, LPE is administered on a wide range of crops, such as green pepper, sweet cherry, strawberries, and tomatoes [12–14]. Exogenously applied LPE delays early senescence while simultaneously accelerating fruit ripening and increasing the half shelf-life of crop plants [12, 15, 16]. LPE’s influence depends on the stage of fruits’ ripening. LPE stimulates ripening in mature fruits; however, LPE interferes with ethylene-production in ripening fruits resulting in an improved structural strength, which consequently extends the shelf life. After 5 days of LPE-treatment, fruits possessed a significantly lower production of ethylene and a diminished level of electrolyte leakage than the controls. The specific role of LPE in aging and senescence might be explained by the inhibitory effect of LPE on phospholipase D, an enhancer of senescence progression [15, 17, 18].

Plant immunity and especially pathogen recognition can be classified into at least two distinct branches. Firstly, the perception of characteristic pathogen-associated molecular components/pattern (PAMP) by the plasma membrane receptor complex FLAGELLIN-INSENSITIVE2 / BRI1-ASSOCIATED RECEPTOR KINASE activates a MAP-kinase signaling cascade that links up-stream signals to downstream targets by phosphorylation and refers to the PAMP-triggered immunity (PTI). FLAGELLIN22 (flg22), a 22 amino acid peptide from within the bacterial flagellin protein, is a crucial PAMP that triggers PTI in plants [19, 20]. Secondly, the perception of bacterial effectors by cytosolic plant receptors triggers a robust defense response, called effector-triggered immunity (ETI) [21, 22]. Plant defense against fungi and other microbial pathogens relies on the induction of both local and systemic resistance [23].

The three phytohormones salicylic acid (SA), jasmonic acid (JA), and ethylene (ET) are the classical immunity signaling agents, that orchestrate defense processes depending on the kind of biotic threat. The local and systemic acquired resistance (SAR) depends on SA action [24, 25]. SA-biosynthesis and signaling are induced after direct exposure to a wide range of biotrophic and hemibiotrophic pathogens, like *Pseudomonas syringae* (*Pst*DC3000). Pathogen-induced SA biosynthesis mainly takes place in chloroplasts catalyzed by *ISOCHORISMATE SYNTHASE 1* (*ICS1*) [26, 27]. Several transcription factors (TFs) have been reported to be directly recruited to the *ICS1* promoter in order to activate its expression. Of these, SYSTEMIC ACQUIRED RESISTANCE DEFICIENT 1 (SARD1) and CALMODULIN-BINDING PROTEIN 60 g are essential for *ICS1* induction and SA accumulation after pathogen perception [28, 29]. The WRKY family members WRKY28 and WRKY46 have been shown to promote the induction of *ICS1* as well, partly in complex with SARD1 [30, 31]. ENHANCED-DISEASE SUSCEPTIBILITY (EDS) 1 and PHYTOALEXIN-DEFICIENT (PAD) 4 contribute to SA synthesis in guard cells [32], form heterodimers, and are regulated by the positive feedback of SA which in turn potentiates SA action. Moreover, the SA-efflux-transporter EDS5, which is localized in the chloroplast envelope, contributes to intracellular SA-homeostasis [33–35]. A hub in SA-signaling is formed by NON-EXPRESSOR OF PATHOGENESIS-RELATED GENES (NPR) 1 and its counteracting paralogs NPR3 and NPR4. These factors act as SA receptors [36, 37], whose function is mediated by members of the TGA family [38, 39] and by the NIM1-INTERACTING (NIMIN) proteins [40]. However, NPR3/4 exert distinct roles in the transcriptional regulation of SA-inducible genes [41] by mediating the action of the transcriptional regulator NPR1 which is considered the ‘master regulator’ of the SAR responses [41, 42]. A feed-forward loop between SA and reactive oxygen species (ROS) production, such as hydrogen peroxide (H_2_O_2_) in the defense response, was reported, and ROS signals are involved both upstream and downstream of SA signaling in response to stress [43]. The majority of intracellular H_2_O_2_ is produced from molecular oxygen by a stepwise reaction via a superoxide anion intermediate which undergoes enzymatic reduction to H_2_O_2_ [44]. In this context, H_2_O_2_ originating in chloroplasts and peroxisomes triggers SA biosynthesis. H_2_O_2_ is essential for the main outputs of the multifaceted defense response comprising transcriptional reprogramming, cell death, and stomatal closure [43]. By contrast, JA and ET signaling promote immunity against necrotrophs such as the fungal pathogen *Alternaria brassicicola* [45–47]*, Botrytis cinerea* [48] or *Cochliobolus miyabeanus* [49]. Moreover, a large number of studies have revealed that the SA signaling pathway prevalently acts antagonistically to ET/JA-mediated signaling [50, 51] in plant defense response.

We analyzed the effect of LPE on plant immunity. LPE-pretreated plants express molecular markers for SA-biosynthesis and signaling. Moreover, in-situ ROS-determination of LPE-treated plants revealed the accumulation of H_2_O_2_, which correlates with the induced expression of oxidative stress markers. We found that LPE-pretreated *Arabidopsis* plants are more resistant to *Pst*DC3000*,* indicating LPE as a plant defense-promoting factor.

## Results

### LPE-treatment induces the expression of SA-signaling and biosynthesis genes

**Fig. 1:**
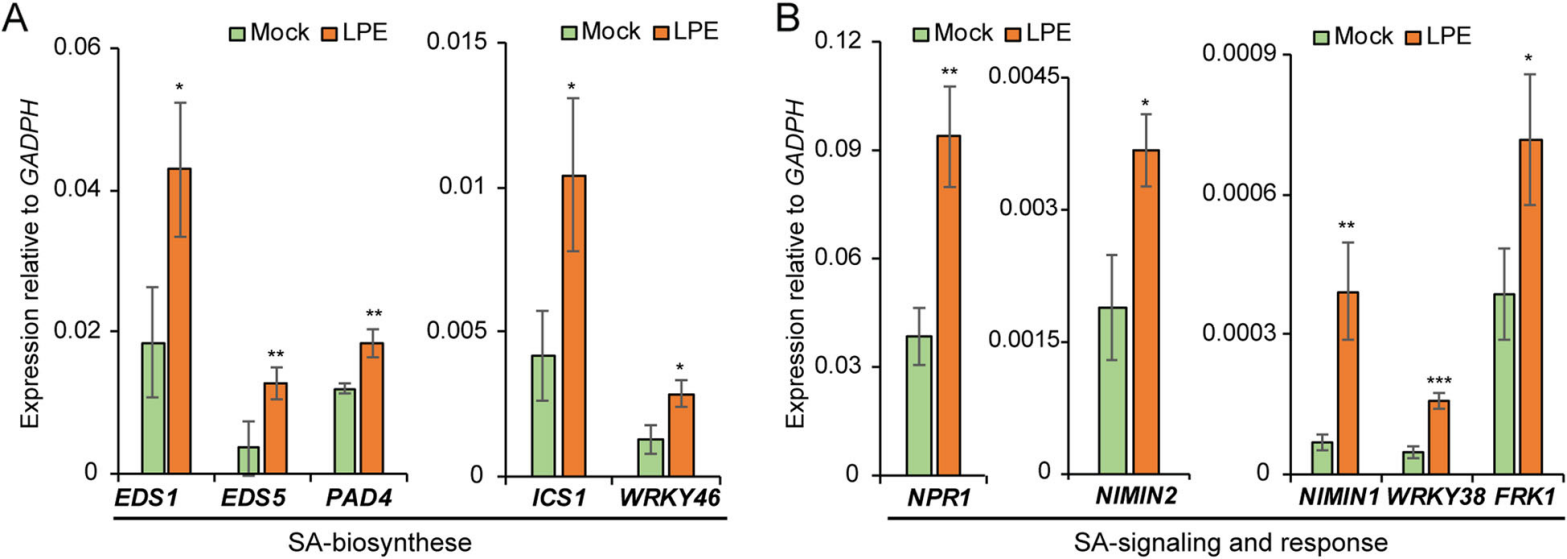
SA-biosynthesis, signaling and response genes are strongly expressed after LPE-treatment. **a** Expression of SA-biosynthesis genes *EDS1, EDS5, PAD4, ICS1* and *WRKY46,* 24 h after LPE-administration. **b** Transcript levels of the SA-signaling component *NPR1, NIMIN1, NIMIN2*, *WRKY38* and of the PAMP/ SA-marker *FRK1,* 24 h post LPE-application. Error bars show ± SEM; statistical significance was analyzed by one-way ANOVA against mock control, *p < 0.05, **p < 0.01. Values indicate the mean of three biological replicates

To evaluate the impact of LPE on plant gene expression, we analyzed the induction of defense genes and immunity markers following LPE-application. We found that several components determining SA-biosynthesis were differentially expressed after LPE-treatment*. ICS1*, which encodes a protein that catalyzes the rate-limiting step in the SA-metabolism for plant defense [26, 27] was more strongly expressed after LPE-treatment than in the mock-treated control (Fig. 1a). In addition, we observed elevated transcript levels of *WRKY46*, which encodes a protein that contributes to *ICS1* expression [31] (Fig. 1a). Moreover, the number of transcripts of *EDS1, EDS5,* and *PAD4* increased in LPE-treated plants (Fig. 1a). The hub in SA-reception and signaling is formed by NPR1 and its counteracting paralogs NPR3 and NPR4. Interestingly, *NPR1* (Fig. 1b), encoding the SA-receptor [36, 52], and the expression of the SA-receptor components, *NIM1-INTERACTING* (*NIMIN*) *1* (Fig. 1b), *NIMIN2* (Fig. 1b) [39, 53] and *WRKY38* (Fig. 1b), were increased following LPE-treatment. However, the expression of *NPR3* and *NPR4* (Fig. 1b), was indistinguishable from the mock-treated control. The transcriptional induction of the PAMP [54] and SA-response marker [55, 56] *FLG22-INDUCED RECEPTOR-LIKE KINASE 1* (*FRK1*) was more strongly induced following LPE-administration (Fig. 1b). This result suggests that LPE activates the immune MAP-kinase cascade thereby initiating plant immunity.

Importantly, key-marker genes for JA/ET-biosynthesis and signaling were found at mock treatment levels. These genes include *OXOPHYTODIENOATE REDUCTASE* (*OPR3*), whose encoded protein catalyzes the rate-limiting step in the JA-biosynthesis, the JA-response gene *VEGETATIVE STORAGE PROTEIN 2* (*VSP2*), and the key-target factors of the synergistic JA/ET-response *PLANT DEFENSIN 1.2A* (*PDF1.2*), *ETHYLENE RESPONSE FACTOR 1* (*ERF1*) and *OCTADECANOID-RESPONSIVE ARABIDOPSIS AP2/ERF 59* (*ORA59*) (Fig. S1) [57–59]. The regular expression of these pivotal factors mediating JA/ET action suggests that LPE does not modulate JA/ET-dependent defense before pathogen-perception.

Altogether, in solely LPE-treated plants, the differential expression of SA-metabolic and SA/PAMP signaling genes, suggests a degree of plant genome priming that supports defense-associated processes and mechanisms.

### Senescence-associated genes are differentially expressed

**Fig. 2:**
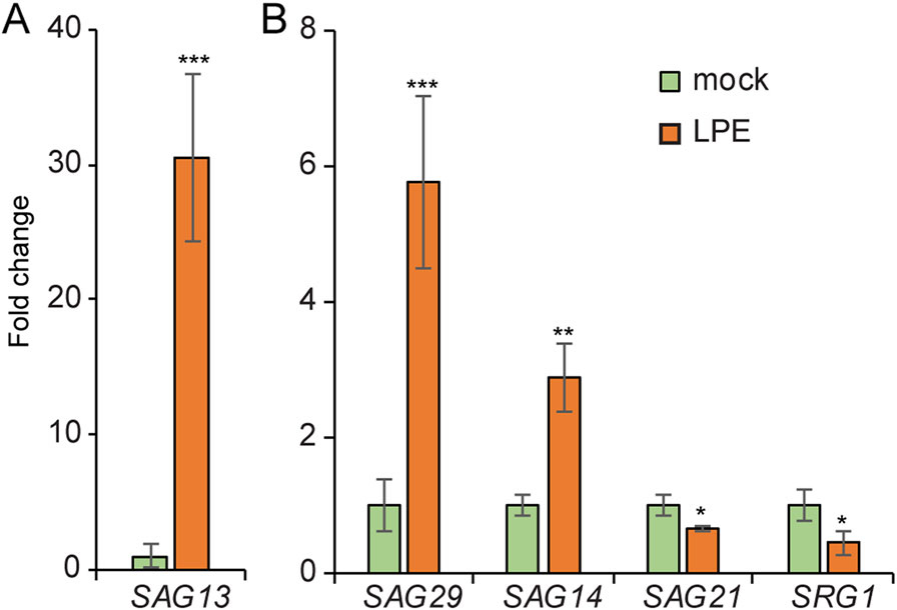
Senescence-associated genes are differentially regulated after LPE-treatment. **a-b** Transcript abundance of the *SENESCENCE ASSOCIATED GENEs* (*SAG*) *13, SAG29, SAG14, SAG21* and the *SENESCENCE-RELATED GENE* (*SRG*) *1*. Depicted is the fold change of the transcript amount relative to *GADPH*. Error bars show ± SEM; statistical significance was analyzed by one-way ANOVA against mock control; *p < 0.05, **p < 0.01, *** *p* < 0.001. Values indicate the mean of three biological replicates

Changes in SA-induced and age-dependent gene expression show a high degree of overlap in genome-wide transcriptomic data sets, that demonstrate the dual role of SA in leaf senescence and plant immunity [60]. Therefore, we analyzed whether the LPE-induced up-regulation of SA-metabolic genes corresponds with the differential expression of genes involved in senescence and aging. *SENESCENCE ASSOCIATED GENE* (*SAG*) *13* was shown to be induced by ROS and to be involved in immunity against necrotrophic and biotrophic pathogens [61]. *SAG29* belongs to the SWEET sucrose efflux transporter family and is strongly expressed in young inflorescent buds but the expression declines in senescent leaves [62]. The transcript abundance of *SAG13* and *SAG29* is strongly increased after LPE-treatment (Fig. 2). Likewise, *SAG14*, a blue copper-binding protein [63], was upregulated (Fig. 2). Yet, *SAG21* [63], involved in oxidative stress tolerance, and the *SENESCENCE-RELATED GENE* (*SRG*) *1* [64] exhibit a reduced transcript quantity following LPE-treatment (Fig. 2). In summary, the differential expression of *SAG*s is in accordance with previous findings concerning the inhibitory effect of LPE on fruit ripening and senescence progression.

### LPE enhances the hydrogen-peroxide accumulation

SA plays a critical role in transcriptional reprogramming, cell-death, and systemic-acquired resistance during defense response. Several lines of evidence indicate that the non-radical ROS hydrogen peroxide H_2_O_2_, originating in chloroplasts and peroxisomes, triggers SA-biosynthesis. Furthermore, SA also promotes ROS production during the early-stages of defense response [43].

**Fig. 3:**
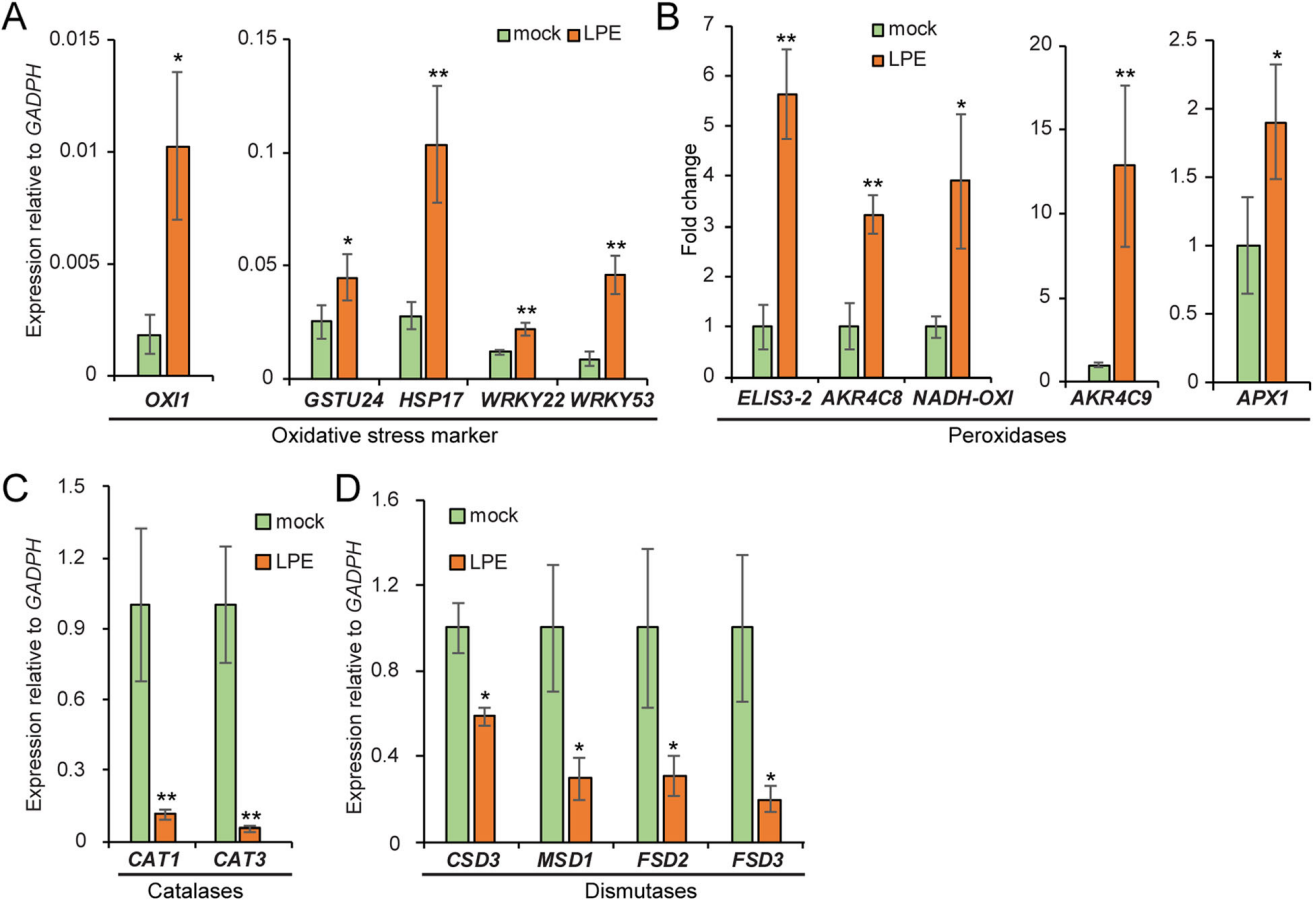
Genes, involved in ROS signaling, production and scavenging, are differentially expressed after LPE-application. **a** Expression of *OXI1, GSTU24, HSP17, WKRY22,* and *WRKY53* deemed as markers for oxidative stress response and H_2_O_2_ accumulation. *WRKY22 and WRKY53* are also shown to be early defence-marker associated with PAMP-triggered immunity. **b** Peroxidase *ELIS3–2, AKR4V8, NADH-OXI, AKRC9* and *APX1* are up-regulated. **c** The transcript abundance of the catalases *CAT1* and *CAT2* is strongly reduced, (**d**) accordingly to the expression of the dismutases *CSD3, MSD1, FSD2* and *FSD3*. **a-d** Error bars show ± SEM; statistical significance was analysed by one-way ANOVA against mock control, *p < 0.05, **p < 0.01. Values indicate the mean of three biological replicates for mock and LPE-treated samples (24 h)

We analyzed whether the induction of key genes involved in oxidative signaling and response to intracellular, cytosolic H_2_O_2_ accumulation correlates with our findings. Thus, we determined the induction of the H_2_O_2_-inducible genes *Arabidopsis thaliana GLUTATHIONE S-TRANSFERASE 24* (*GSTU24*) [65, 66], involved in the glutathione-related signaling during enhanced H_2_O_2_ metabolism. Besides, we analyzed the small heat shock gene *HSP20-LIKE CHAPERONES SUPERFAMILY PROTEIN* (*HSP17.6A*) [67] and the *OXIDATIVE SIGNAL-INDUCIBLE 1* (*OXI1*) that encodes a serine/threonine kinase induced in response to a wide range of H_2_O_2_ -generating stimuli [68]. Compared to the mock-treated control all three key genes are significantly upregulated after LPE-treatment (Fig. 3a). Likewise, *WRKY22*, an early-immunity marker [54] previously shown to have an elevated expression in response to H_2_O_2_ [69], and its H_2_O_2_-inducible upstream regulator *WRKY53* [70], are more highly expressed 24 h after LPE-application (Fig. 3a). In summary, our expression data suggests that LPE primes the plant genome and promotes genes involved in oxidative stress and H_2_O_2_ accumulation.

Plants have a complex antioxidant system for maintaining the homeostasis of ROS. In general, this system can be divided into enzymatic and non-enzymatic ROS-scavenging mechanisms [71]. Thus, we determined the expression levels of genes involved in the metabolism and catabolism of different sorts of ROS. Three types of antioxidative enzymes, superoxide dismutases (SOD), catalases (CAT) and peroxidases, play a major role in keeping superoxide radicals and H_2_O_2_ at steady-state levels under non-stress conditions [72]. Oxidoreductases, known formerly as oxidases, reductases, dehydrogenases and peroxidases, predominately generate ROS in various cell compartments. The oxidoreductase encoding genes *CINNAMYL-ALCOHOL DEHYDROGENASE B2* (*ELIS3–2*) [73], two *CHLOROPLASTIC ALDO-KETO REDUCTASEs* (*AKR4C8* and *AKR4C9*) [74], a *NADH:UBIQUINONE/PLASTOQUINONE OXIDOREDUCTASE* (*NADH-OXI*) and *ASCORBATE PEROXIDASE 1* (*APX1*) [75] exhibited higher transcript levels after LPE-treatment (Fig. 3b). By contrast, *CAT1* and *CAT3,* which encode proteins that degrade H_2_O_2_ [76], are strongly downregulated (Fig. 3c). SODs are a diverse set of enzymes that generate H_2_O_2_ thereby containing either iron (Fe), manganese (Mn), or copper/zinc (Cu/Zn), and are present in the cytosol, chloroplasts, mitochondria and peroxisomes [77, 78]. We found a reduced expression of *SOD*s (*CSD3, MSD1, FSD2, FSD3*) after LPE-treatment (Fig. 3d). In summary, genes that contribute to the antioxidant system for maintaining the homeostasis of ROS are differentially regulated after LPE-application thereby suggesting an altered ROS titer.

**Fig. 4:**
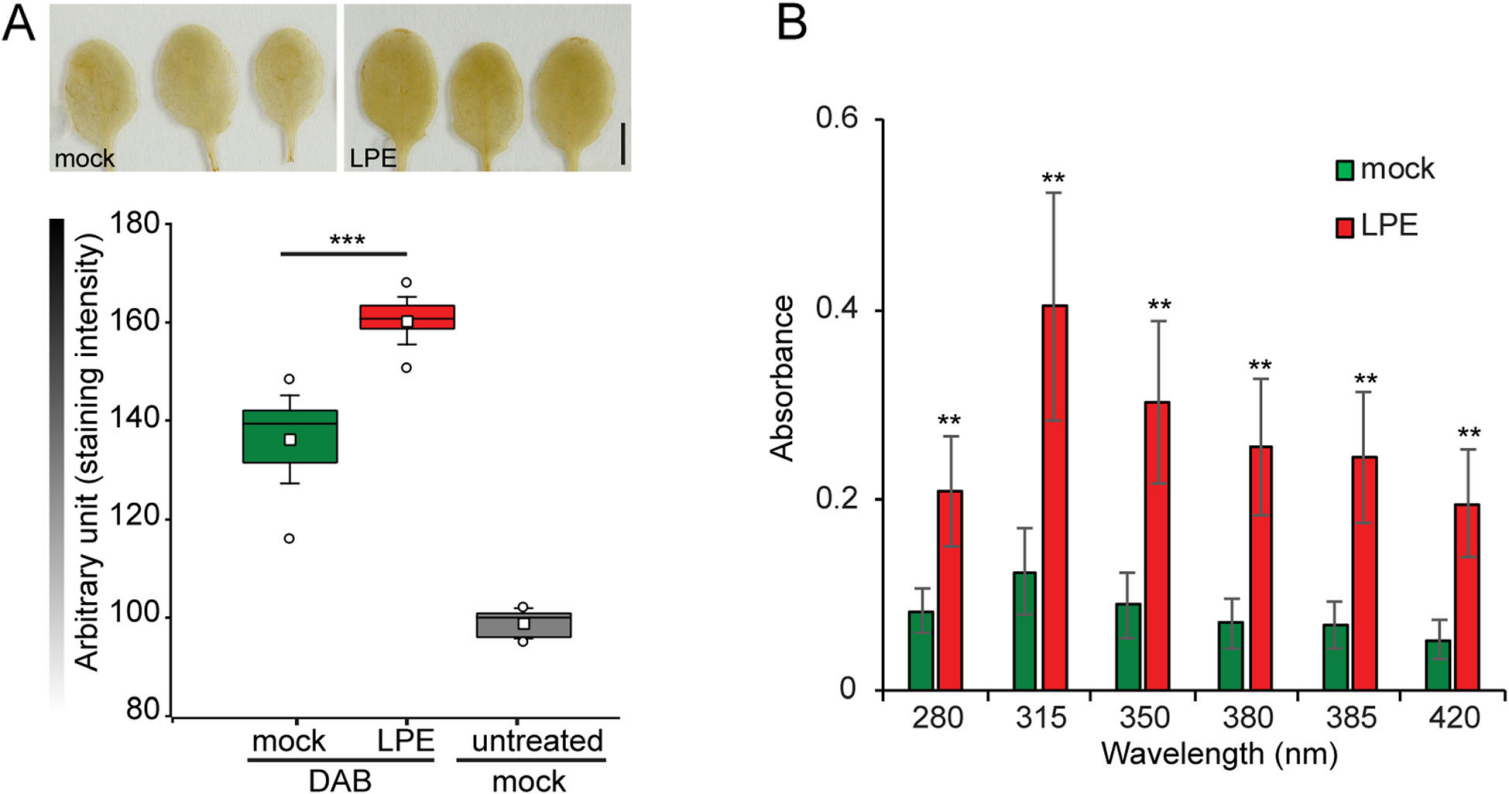
Hydrogen-peroxide levels are elevated following LPE-application. **a** Evaluation of H_2_O_2_ levels by 3,3′- diaminobenzidine staining (DAB) 24 h after LPE-treatment compared to the mock-treated control. Scale bar = 5 mm. Boxes represent the 25th and 75th percentiles, and the inner rectangle highlights the median, whiskers show the SEM, and outliers are depicted by dots (Min/max range). Statistical significance was analysed by one-way ANOVA; asterisks indicate significant difference compared to the mock/DAB control, *** *p* < 0.001. Representative images of three biological replicates are shown. **b** Absorption spectra of H_2_O_2_ reaction with potassium iodide (KI 1 M) in 0.1% TCA spectrophotometrically determined at different wavelengths between 280 and 420 nm

Therefore, we raised the question of whether the differential expression of these genes corresponds to deviating in situ ROS levels. To assess the accumulation of the intracellular ROS H_2_O_2_ 24 h after LPE-application, we carried out an in situ 3,3′-diaminobenzidine (DAB) staining of mock and LPE-treated plants (Fig. 4a). In the untreated control without applied DAB, the staining value is approximately 165 arbitrary units and was set as the default unstained level. After DAB staining, mock-treated plants showed a weak staining intensity of 125 compared to strong staining of 100 in LPE-treated plants. This staining difference indicates that LPE triggers H_2_O_2_ accumulation *in planta*. To validate these results, we quantitatively determined H_2_O_2_ concentrations based on the spectrophotometric analysis of potassium iodide oxidation [79]. The absorbance at different wavelengths between 280 and 420 nm was consistently elevated compared to the mock-treated controls, demonstrating higher ROS levels (Fig. 4b). Consequently, our results indicate that LPE changes the transcriptomic profile of antioxidant genes thereby influencing ROS homeostasis.

### LPE interferes with the oxidative burst following PAMP perception

**Fig. 5:**
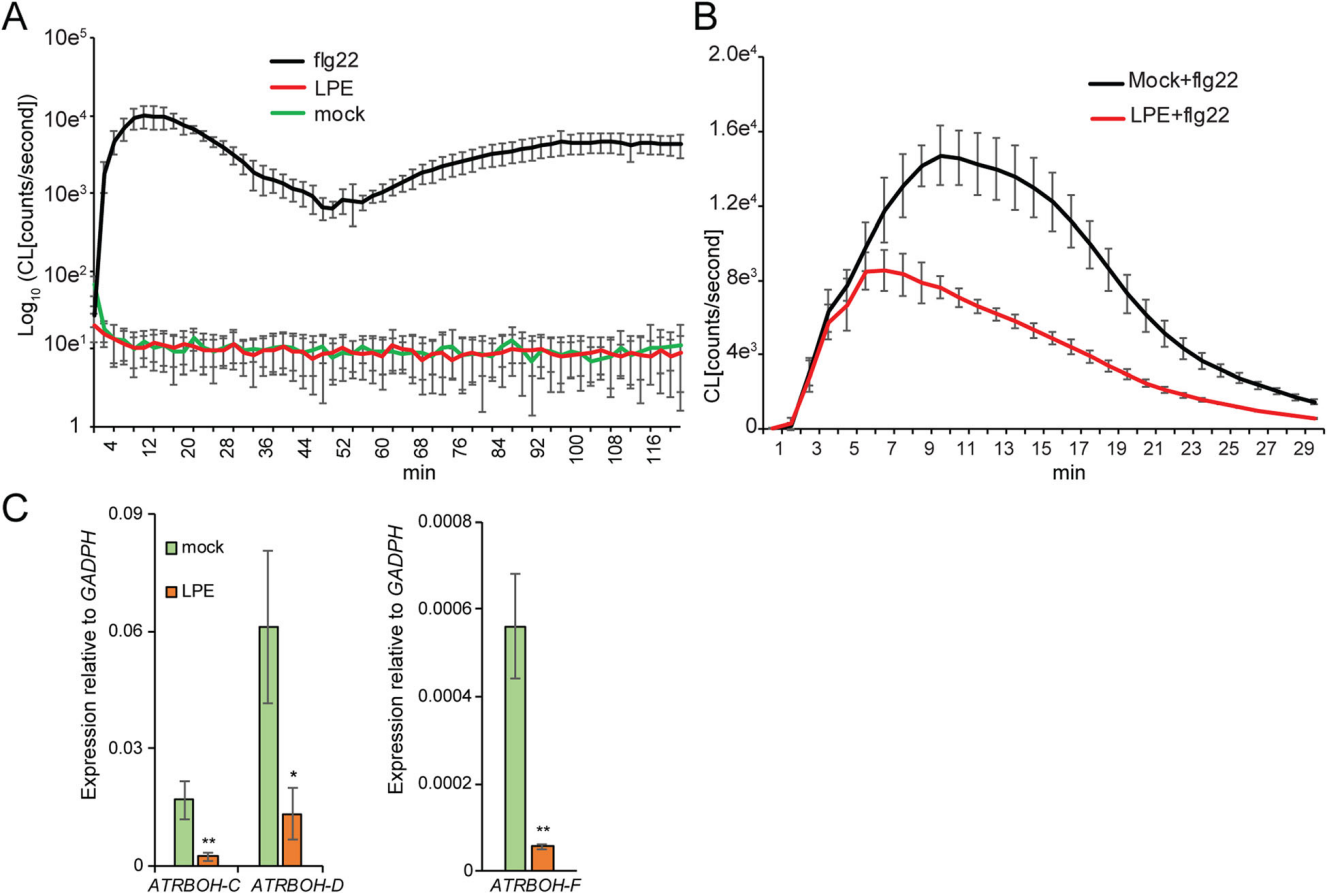
LPE-application compromises the ROS efflux in response to PAMP-perception. **a** Depicted is the ROS-burst kinetic of LPE-treated plants (50 mg/l) compared to flg22 and mock-treated control for 120 min. Values indicate the Log_10_mean ± SE 5-week old plants. **b** ROS-burst assay of LPE-pretreated plants followed by flg22-treatment (1 μM) for 30 min. Shown is the mean ± SE (B), *n* = 36 (3 biological replicates). **c** The expression of the *NADPH oxidases*, also referred to as respiratory burst oxidases (*RBOHs*), is reduced 24 h after LPE-administration. Error bars show ± SEM; statistical significance was analysed by one-way ANOVA against mock control, *p < 0.05, **p < 0.01. Values indicate the mean of three biological replicates for mock and LPE-treated samples (24 h)

Furthermore, we analyzed whether LPE triggers the ROS-burst, one of the first defense reactions in plants after the perception of pathogen-associated molecular pattern (PAMP) [20]. In the ROS-burst assay, as a positive control, we applied the PAMP flg22 (100 nm), which was demonstrated to massively increase the ROS-burst [20]. However, the applied LPE (50 mg/l) did not trigger ROS-burst and the outcome was indistinguishable from the mock-treated control (Fig. 5a). Subsequently, we examined whether LPE pretreated plants show a difference in the ROS-burst after flg22 perception. Interestingly, the ROS-burst was consistently diminished in 3 biological replicates after flg22 application compared to the mock-pretreated control (Fig. 5b). This finding suggests that LPE interferes with the ROS-burst machinery after PAMP perception.

The predominant source of apoplastic H_2_O_2_ is catalyzed by apoplastic peroxidases and NADPH oxidases also referred to as respiratory burst oxidases (RBOHs) [80]. RBOH-D and RBOH-F were found to be required for a full oxidative burst in response to avirulent strains of *Pst*DC3000 [81]. We found the main members *AtRBOH-C, AtRBOH-D,* and *AtRBOH–F* being downregulated after LPE-treatment (Fig. 5c). This downregulation aligns with the attenuated ROS-burst observed after LPE + flg22-treatment compared to the mock+flg22 control. Taken together, our results show that LPE enhances the H_2_O_2_ accumulation over 24 h, which correlates with the expression of markers for oxidative signaling and elevated H_2_O_2_ levels. Yet, the burst of H_2_O_2_ and further members of the ROS family, like singlet oxygen and superoxide anions, is diminished by LPE after PAMP perception. This finding identifies LPE as a negative regulator of apoplastic ROS production and burst.

### LPE-application increases the resistance of *Arabidopsis thaliana* against *Pseudomonas syringae*

**Fig. 6:**
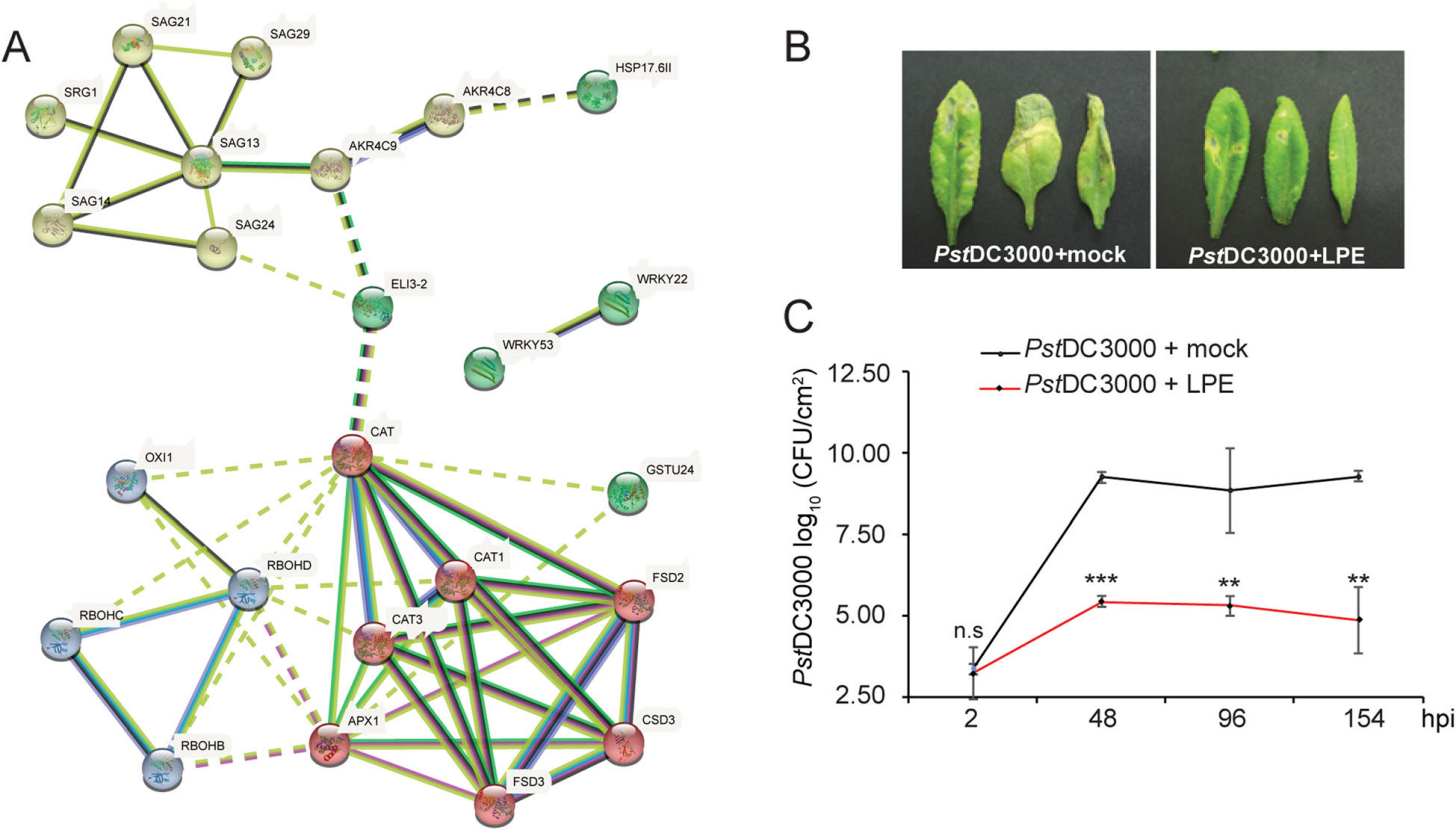
Exogenously applied LPE increases plant resistance to *Pseudomonas syringae.* **a** Differentially regulated genes after LPE-treatment were pooled and used to generate a functional protein interaction network by the use of STRING (version 10.0) within the Cytoscape environment. Interconnected clusters contain targets involved in senescence (yellow), ROS metabolism (blue, red) and MAP kinase signaling (green). **b** Overview of four-week-old WT plants, after mock- and LPE-pretreatment, followed by *Pst*DC3000 spray-inoculation. **c** WT (Col) plant, were challenged with *Pst*DC3000 after LPE- or mock-pretreatment. Three biological replicates of ten plants (*n* = 30) were spray-inoculated with a bacterial suspension of OD_600_ = 0.2, the density of colony-forming units (CFU) was analyzed 2, 48, 96 and 154 h post-inoculation (hpi). Statistical significance was analyzed by one-way ANOVA, asterisks indicate significant difference, n.s., non-significant; ** *p* ≤ 0.01; ***p ≤ 0.001. Error bars show ± SEM

To study the effect of LPE on plant immunity, we treated four to five-week-old *A. thaliana* plants (ecotype Columbia) with LPE, 24 h before inoculation (Fig. 6b, c) and subsequently challenged these plants with *Pst*DC3000. Two hours after spray inoculation, the bacterial proliferation levels in LPE-pretreated plants corresponded to those in the control plants indicating that stomatal immunity, and hence, bacterial leaf-accessibility was not affected by the applied LPE. However, the bacteria proliferation was consistently diminished from 48 to 154 hpi in LPE-pretreated plants compared to the mock-pretreated control, thereby indicating a reduced susceptibility of *Arabidopsis* after LPE-application. In summary, these results demonstrate that administered LPE increases resistance against the hemibiotrophic pathogens *Ps*t*DC3000*.

## Discussion

We discovered that the phospholipid LPE, a regulator of early senescence in plants, contributes to plant innate immunity. LPE-pretreated plants exhibit enhanced resistance against *PstDC3000*.

ROS can be produced in different organelles in response to a broad range of specific environmental conditions [82]. In this regard, ROS accumulation enables the activation of a multitude of signaling cascades, which results either in acclimation or in cell death depending on the biotic/abiotic stress. Whether ROS would serve as signaling molecules or could cause oxidative damage to the tissues depends on the delicate equilibrium between ROS production, and their scavenging. In mitochondria and chloroplasts, generated ROS is an unavoidable by-product of bioenergetic processes. Within chloroplasts, triplet state excited chlorophylls, and the electron transfer chain are major sites of ROS production [83]. We found that LPE-application enhances the level of hydrogen peroxide, which goes along with the differential expression of cytosolic markers for oxidative stress, H_2_O_2_ production and scavenging, as well as senescence-associated genes. The dismutation of two molecules of H_2_O_2_ into water and oxygen is catalyzed by CATs. We found that *CAT1* and *CAT3* were downregulated after LPE treatment which may explain the increase in H_2_O_2_ levels. SODs, which generate H_2_O_2_, showed a reduced expression that might be caused by a negative feedback loop on *SOD* expression at high H_2_O_2_ levels.

Following the downregulation of plasma membrane-localized respiratory burst oxidase genes, which lead to the catalysis of apoplastic H_2_O_2_, the ROS-burst was diminished after PAMP perception in LPE-pretreated plants. This result refers to a reduced apoplastic H_2_O_2_ titer. However, we found markers for cytosolic H_2_O_2_ accumulation strongly expressed in agreement with high observed H_2_O_2_ measurements. Eventually, our findings support the notion that LPE triggers the increase of cytosolically-localized H_2_O_2_, but diminishes the amount of apoplastic ROS. The obtained results suggest that the LPE-mediated increase of intracellular H_2_O_2_ levels favors plant immunity over the release of ROS as an immediate defense response.

Differentially regulated genes (DEGs) after LPE-treatment were pooled and used to generate a functional protein interaction network by the use of STRING [84]. The network revealed that the DEGs can be grouped in functional clusters that are interconnected (Fig. 6a). This result shows that LPE exerts transcriptional regulation on molecular components that concomitantly contribute to ROS homeostasis and senescence.

The early immunity markers *WRKY22* and *FRK1* are induced by the PAMP-triggered MAP-kinase signaling cascade (MEKK1, MKK4/MKK5, and MPK3/MPK6) [54] that function downstream of the flagellin receptor FLS2, a leucine-rich-repeat (LRR) receptor kinase. The expression of these marker genes indicates the activation of the MAP-kinase cascade, which results in the promotion of the SA signaling cascade and the accumulation of reactive oxygen species [55]. Eventually, the activation of this MAP-kinase cascade confers resistance to bacterial pathogens [85, 86], which is in accordance with our findings.

Damage-associated molecular patterns (DAMPs) are tissue-derived alarm signals that trigger cellular signaling cascades which prevalently initiate defense responses. DAMPs are deemed as any molecules that are usually not exposed to cells under non-stress conditions, such as cell wall components, nucleic acid fragments, peptides, extracellular ATP, and further components [87]. Importantly, DAMPs are derived from the injured organism in which the response cascade will be initiated. Thus, LPE, as a general plant cell-membrane component, can be taken into account as a DAMP that triggers defense-associated signaling pathways.

## Conclusion

Taken all together, our results indicate that LPE acts as an immunity-promoting agent that activates a wide-range of defense-related-traits associated with SA-metabolism and H_2_O_2_ turnover. This outcome suggests that SA/ROS homeostasis is a crucial element for the LPE-enhanced plant immunity against hemibiotrophs. These findings open up the possibility to apply LPE in farming, not only to delay senescence of crops but also to improve their resistance to biotic threats under inhospitable conditions, and further work on that aspect is warranted.

## Methods

### Plant materials

Seeds of *Arabidopsis thaliana* ecotype Col-0 were obtained from the European Arabidopsis Stock Center (N1093). *Arabidopsis* plants were grown in a growth chamber (Percival) at 22 °C, 70% relative humidity with 16 h illumination.

### Pathogen inoculation

Four- to five-week-old *A. thaliana* plants, grown in soil and bottom-irrigated, were sprayed with either 250 ppm Tween 80 (mock-solution) or 50 ppm LPE (50 mg/l) [16] in 250 ppm Tween 80 solution 24 h before pathogen inoculation. Afterward, plants were covered by a hood and kept in the growth chamber at 22 °C. *Pseudomonas syringae pv. tomato* (Pst) DC3000 strains were grown for 24 h at 28 °C on King Agar B media plates (Sigma 60,786) with 50 mg/l rifampicin. *Pst* DC3000 were regained from plates in 10 mM MgCl_2_. Plants were spray-inoculated with *Pst*DC3000 at OD_600_ = 0.2 in 10 mM MgCl_2_ containing 0.04% Silwet L-77 and sampled 2 h, 48 h, 96 h and 154 after inoculation. To determine the level of colonization (colony-forming units (CFU)), a total of 30 plants (3 biological replicates) were sampled by taking 3 leaf discs per plant and the bacterial titers of the plant samples were determined by conducting a dilution series at 28 °C on King Agar B plates with 50 mg/l rifampicin [88].

### RNA extraction and qRT-PCR

RT-PCR was performed to analyze expression levels of individual marker genes. Plants were grown on on plates [half-strength Murashige and Skoog (MS, Sigma M6899), 0.5% sucrose (sigma S5016), 1% agar (sigma A1296), and 0.5% MES (sigma M8250), pH adjusted to 5.7 with KOH] at 23 °C in long-day conditions. Total RNA from LPE (50 mg/l) and mock-treated 14-days old seedlings was extracted using the easy-spin™ Total RNA Extraction Kit (iNtRON BIOTECHNOLOGY).

An amount of 5 μg of total RNA was reverse transcribed using SuperScript II reverse transcriptase (Invitrogen, Carlsbad, CA). The RT-qPCR analysis was performed by the use of an Applied Biosystems 7500 Real-Time PCR system™ (Applied Biosystems, Foster City, CA). An SYBR green PCR master mix (Applied Biosystems, Warrington, UK) was used. Data generated from duplicates of at least three biological replicates (biorep) were averaged. The relative increase or decrease of mRNA abundance between samples was determined by comparing the threshold cycle values. Oligo-nucleotides used to determine the transcript levels can be found in Table S1.

### Histochemical staining and ROS-burst assay

In situ detection of H_2_O_2_ was performed from LPE (50 mg/l) and mock-treated 4 weeks old plants by the use of 3,3′-diaminobenzidine (DAB) histochemical staining, as previously described [89]. Experiments were repeated three times with similar outcomes and shown is a representative result. Detection of ROS-burst after LPE, mock and flg22 (100 nM)-treatment was carried out as previously described [90]. In brief, ROS-burst was measured using a luminal-based assay. Leaf discs (1/4 in. in diameter) of LPE and mock-pretreated plants were incubated overnight in a white 96-well plate (Costar, Fisher Scientific) containing sterile water. After 24 h, the water was replaced by 100 μl of the elicitation solution (34 μg/ml luminol, 20 μg/ml horseradish peroxidase and 1 mM flg22). Luminescence was measured using the GLOMAX 96 MICROPLATE LUMINOMETER, and signal integration time was 0.5 s. Data of three bioreps (consisting of 12 technical replicates per biorep) were acquired, and the average value is presented.

### Spectrophotometrical analysis of H_2_O_2_ levels

The measurement was carried out by following the instructions of Nounjan et al., 2012 [79] with some modifications. In brief, 14 day-old Arabidopsis seedlings (50 mg) were homogenized and subsequently taken up in 1.25 ml of solution containing 1.0 ml trichloroacetic acid (TCA) (0.1% w:v) and 0.25 ml potassium phosphate buffer (10 mM). The suspension was centrifuged (12,000 rpm, 15 min, 4C). Each time 500 μl of the supernatant were added to either 200 μl potassium-jodide (1 M) or to 200 μl water, respectively. Subsequently, 200 μl of each tube were placed in UV-microplate wells and left at room temperature for 1 h. Samples and blanks were analysed in triplicates. The oxidation of potassion iodid was determined by the Epoch-spectrophotometer from BioTeck at 280, 315, 350, 380, 385 and 420 nm.

### Statistical analysis

Statistical significance was calculated based on one-way ANOVA with Tukey post-test. Different letters above bars indicate significant differences, *p* < 0.05. Samples sharing letters are not significantly different. Asterisks indicate significant differences n.s., non-significant, *p < 0.05, **p < 0.01, ***p < 0.001.

## Supplementary Information

**Additional file 1: Figure S1.** Genes, involved in JA/ET metabolism and SA signalling repression, are not differentially expressed after LPE-treatment.

**Additional file 2: Table S1.** Oligo-nucleotides used to determine the transcript levels.

## Data Availability

The data that support the findings of this study are available from the corresponding author upon reasonable request.

## Abbreviations

LPE: *Lyso*-phosphatidylethanolamine
SA: Salicylic acid
ET: Ethylene
JA: Jasmonic acid
ROS: Reactive oxygen species
H_2_O_2_: Hydrogen peroxide
MAP: Mitogen-activated protein
flg22: Flagellin22
PAMP: Pathogen-associated molecular pattern
PTI: PAMP-triggered immunity
ETI: Effector-triggered immunity
DEGs: Differentially regulated genes
*ICS1*: *ISOCHORISMATE SYNTHASE 1*
*SARD1*: *SYSTEMIC ACQUIRED RESISTANCE DEFICIENT 1*
*EDS*: *ENHANCED-DISEASE SUSCEPTIBILITY*
*PAD*: *PHYTOALEXIN-DEFICIENT*
*NPR*: *NON-EXPRESSOR OF PATHOGENESIS–RELATED GENES*
*NIMIN*: *NIM1-INTERACTING*
*FRK1*: *FLAGELLIN22-INDUCED RECEPTOR-LIKE KINASE 1*
*OPR3*: *Oxoxphytodienoate reductase 3*
*VSP2*: *VEGETATIVE STORAGE PROTEIN 2*
*PDF1.2*: *Plant defensin 1.2A*
*ERF1*: *Ethylene response factor 1*
*ORA59*: *OCTADECANOID-RESPONSIVE ARABIDOPSIS 59*
*GSTU24*: *Arabidopsis thaliana GLUTATHIONE S-TRANSFERASE 24*
*HSP17.6A*: *HEAT SHOCK PROTEIN20-LIKE CHAPERONES SUPERFAMILY PROTEIN*
*OXI1*: *OXIDATIVE SIGNAL-INDUCIBLE 1*
*SAG*: *SENESCENCE-ASSOCIATED GENE*
*SRT*: *SENESCENCE-RELATED GENE*
*ELIS3–2*: *ELICITOR-ACTIVATED GENE 3–2*
*AKR*: *ALDO-KETO REDUCTASE*
*NADH-OXI*: *NADH:UBIQUINONE/PLASTOQUINONE OXIDOREDUCTASE*
*APX1*: *ASCORBATE PEROXIDASE 1*
*CAT*: *CATALASE*
*CSD*: *COPPER/ZINC SUPEROXIDE DISMUTASE 1*
*MSD*: *MANGANESE SUPEROXIDE DISMUTASE 1*
*FSD*: *IRON SUPEROXIDE DISMUTASE 1*
*ATRBOH*: *RESPIRATORY BURST OXIDASE HOMOLOG*
DAB: 3,3′-diaminobenzidine
Pst*DC3000*: *Pseudomonas syringae pv. tomato*
Col-0: Columbia
biorep: biological replicate

## References

[1] Delude C, Moussu S, Joubes J, Ingram G, Domergue F (2016). Plant surface lipids and epidermis development. Subcell Biochem.

[2] Siebers M, Brands M, Wewer V, Duan Y, Holzl G, Dormann P (2016). Lipids in plant-microbe interactions. Biochim Biophys Acta.

[3] Mamode Cassim A, Gouguet P, Gronnier J, Laurent N, Germain V, Grison M (2019). Plant lipids: key players of plasma membrane organization and function. Prog Lipid Res.

[4] Lee SM, Suh S, Kim S, Crain RC, Kwak JM, Nam HG (1997). Systemic elevation of phosphatidic acid and lysophospholipid levels in wounded plants. Plant J.

[5] Canonne J, Froidure-Nicolas S, Rivas S (2011). Phospholipases in action during plant defense signaling. Plant Signal Behav.

[6] Lee OR, Kim SJ, Kim HJ, Hong JK, Ryu SB, Lee SH (2010). Phospholipase a(2) is required for PIN-FORMED protein trafficking to the plasma membrane in the Arabidopsis root. Plant Cell.

[7] Lee HY, Bahn SC, Shin JS, Hwang I, Back K, Doelling JH (2005). Multiple forms of secretory phospholipase A2 in plants. Prog Lipid Res.

[8] Lee S, Hirt H, Lee Y (2001). Phosphatidic acid activates a wound-activated MAPK in Glycine max. Plant J.

[9] Hong JH, Chung GH, Cowan AK (2009). Lyso-phosphatidylethanolamine-enhanced phenylalanine ammonia-lyase and insoluble acid invertase in isolated radish cotyledons. Plant Growth Regul.

[10] Way HM, Kazan K, Mitter N, Goulter KC, Birch RG, Manners JM (2002). Constitutive expression of a phenylalanine ammonia-lyase gene from Stylosanthes humilis in transgenic tobacco leads to enhanced disease resistance but impaired plant growth. Physiol Mol Plant P.

[11] Roitsch T, Balibrea ME, Hofmann M, Proels R, Sinha AK (2003). Extracellular invertase: key metabolic enzyme and PR protein. J Exp Bot.

[12] Farag KM, Palta JP (1993). Use of Lysophosphatidylethanolamine, a natural lipid, to retard tomato leaf and fruit senescence. Physiol Plant.

[13] Amaro AL, Almeida DPF (2013). Lysophosphatidylethanolamine effects on horticultural commodities: a review. Postharvest Biol Tec.

[14] Ozgen M, Serce S, Akca Y, Hong JH (2015). Lysophosphatidylethanolamine (LPE) improves fruit size, color, quality and phytochemical contents of sweet cherry c.v. '0900 Ziraat'. Korean J Hortic Sci.

[15] Ryu SB, Karlsson BH, Ozgen M, Palta JP (1997). Inhibition of phospholipase D by lysophosphatidylethanolamine, a lipid-derived senescence retardant. Proc Natl Acad Sci U S A.

[16] Hong JH, Chung G, Cowan AK (2009). Delayed leaf senescence by exogenous lyso-phosphatidylethanolamine: towards a mechanism of action. Plant Physiol Biochem.

[17] Ryu SB, Wang X (1995). Expression of phospholipase D during Castor bean leaf senescence. Plant Physiol.

[18] Wang X (2005). Regulatory functions of phospholipase D and phosphatidic acid in plant growth, development, and stress responses. Plant Physiol.

[19] Bigeard J, Colcombet J, Hirt H (2015). Signaling mechanisms in pattern-triggered immunity (PTI). Mol Plant.

[20] Felix G, Duran JD, Volko S, Boller T (1999). Plants have a sensitive perception system for the most conserved domain of bacterial flagellin. Plant J.

[21] Deslandes L, Rivas S (2012). Catch me if you can: bacterial effectors and plant targets. Trends Plant Sci.

[22] Mackey D, Holt BF, Wiig A, Dangl JL (2002). RIN4 interacts with pseudomonas syringae type III effector molecules and is required for RPM1-mediated resistance in Arabidopsis. Cell.

[23] Durrant WE, Dong X (2004). Systemic acquired resistance. Annu Rev Phytopathol.

[24] Chen Z, Silva H, Klessig DF (1993). Active oxygen species in the induction of plant systemic acquired resistance by salicylic acid. Science.

[25] Gaffney T, Friedrich L, Vernooij B, Negrotto D, Nye G, Uknes S (1993). Requirement of salicylic acid for the induction of systemic acquired resistance. Science.

[26] Strawn MA, Marr SK, Inoue K, Inada N, Zubieta C, Wildermuth MC (2007). Arabidopsis isochorismate synthase functional in pathogen-induced salicylate biosynthesis exhibits properties consistent with a role in diverse stress responses. J Biol Chem.

[27] Wildermuth MC, Dewdney J, Wu G, Ausubel FM (2001). Isochorismate synthase is required to synthesize salicylic acid for plant defence. Nature.

[28] Wang L, Tsuda K, Truman W, Sato M, Nguyenle V, Katagiri F (2011). CBP60g and SARD1 play partially redundant critical roles in salicylic acid signaling. Plant J.

[29] Zhang Y, Xu S, Ding P, Wang D, Cheng YT, He J (2010). Control of salicylic acid synthesis and systemic acquired resistance by two members of a plant-specific family of transcription factors. Proc Natl Acad Sci U S A.

[30] Wang X, Gao J, Zhu Z, Dong X, Wang X, Ren G (2015). TCP transcription factors are critical for the coordinated regulation of isochorismate synthase 1 expression in Arabidopsis thaliana. Plant J.

[31] van Verk MC, Bol JF, Linthorst HJ (2011). WRKY transcription factors involved in activation of SA biosynthesis genes. BMC Plant Biol.

[32] Zheng XY, Zhou M, Yoo H, Pruneda-Paz JL, Spivey NW, Kay SA (2015). Spatial and temporal regulation of biosynthesis of the plant immune signal salicylic acid. Proc Natl Acad Sci U S A.

[33] Nawrath C, Heck S, Parinthawong N, Metraux JP (2002). EDS5, an essential component of salicylic acid-dependent signaling for disease resistance in Arabidopsis, is a member of the MATE transporter family. Plant Cell.

[34] Serrano M, Wang B, Aryal B, Garcion C, Abou-Mansour E, Heck S (2013). Export of salicylic acid from the chloroplast requires the multidrug and toxin extrusion-like transporter EDS5. Plant Physiol.

[35] Yamasaki K, Motomura Y, Yagi Y, Nomura H, Kikuchi S, Nakai M (2013). Chloroplast envelope localization of EDS5, an essential factor for salicylic acid biosynthesis in Arabidopsis thaliana. Plant Signal Behav.

[36] Wu Y, Zhang D, Chu JY, Boyle P, Wang Y, Brindle ID (2012). The Arabidopsis NPR1 protein is a receptor for the plant defense hormone salicylic acid. Cell Rep.

[37] Fu ZQ, Yan S, Saleh A, Wang W, Ruble J, Oka N (2012). NPR3 and NPR4 are receptors for the immune signal salicylic acid in plants. Nature.

[38] Zhou JM, Trifa Y, Silva H, Pontier D, Lam E, Shah J (2000). NPR1 differentially interacts with members of the TGA/OBF family of transcription factors that bind an element of the PR-1 gene required for induction by salicylic acid. Mol Plant Microbe Interact.

[39] Despres C, DeLong C, Glaze S, Liu E, Fobert PR (2000). The Arabidopsis NPR1/NIM1 protein enhances the DNA binding activity of a subgroup of the TGA family of bZIP transcription factors. Plant Cell.

[40] Hermann M, Maier F, Masroor A, Hirth S, Pfitzner AJ, Pfitzner UM (2013). The Arabidopsis NIMIN proteins affect NPR1 differentially. Front Plant Sci.

[41] Ding Y, Sun T, Ao K, Peng Y, Zhang Y, Li X (2018). Opposite roles of salicylic acid receptors NPR1 and NPR3/NPR4 in transcriptional regulation of plant immunity. Cell.

[42] Balint-Kurti P (2019). The plant hypersensitive response: concepts, control and consequences. Mol Plant Pathol.

[43] Herrera-Vasquez A, Salinas P, Holuigue L (2015). Salicylic acid and reactive oxygen species interplay in the transcriptional control of defense genes expression. Front Plant Sci.

[44] Dietz KJ, Turkan I, Krieger-Liszkay A (2016). Redox- and reactive oxygen species-dependent signaling into and out of the photosynthesizing chloroplast. Plant Physiol.

[45] Thomma BP, Nelissen I, Eggermont K, Broekaert WF (1999). Deficiency in phytoalexin production causes enhanced susceptibility of Arabidopsis thaliana to the fungus Alternaria brassicicola. Plant J.

[46] Thomma B, Eggermont K, Penninckx I, Mauch-Mani B, Vogelsang R, Cammue BPA (1998). Separate jasmonate-dependent and salicylate-dependent defense-response pathways in arabidopsis are essential for resistance to distinct microbial pathogens. Proc Natl Acad Sci U S A.

[47] Thomma BP, Eggermont K, Tierens KF, Broekaert WF (1999). Requirement of functional ethylene-insensitive 2 gene for efficient resistance of Arabidopsis to infection by Botrytis cinerea. Plant Physiol.

[48] Li N, Han X, Feng D, Yuan D, Huang LJ (2019). Signaling crosstalk between salicylic acid and ethylene/jasmonate in plant defense: do we understand what they are whispering?. Int J Mol Sci..

[49] Volz R, Park JY, Kim S, Park SY, Harris W, Chung H (2020). The rice/maize pathogen Cochliobolus spp. infect and reproduce on Arabidopsis revealing differences in defensive phytohormone function between monocots and dicots. Plant J.

[50] Robert-Seilaniantz A, Grant M, Jones JD (2011). Hormone crosstalk in plant disease and defense: more than just jasmonate-salicylate antagonism. Annu Rev Phytopathol.

[51] Van der Does D, Leon-Reyes A, Koornneef A, Van Verk MC, Rodenburg N, Pauwels L (2013). Salicylic acid suppresses jasmonic acid signaling downstream of SCFCOI1-JAZ by targeting GCC promoter motifs via transcription factor ORA59. Plant Cell.

[52] Cao H, Glazebrook J, Clarke JD, Volko S, Dong XN (1997). The Arabidopsis NPR1 gene that controls systemic acquired resistance encodes a novel protein containing ankyrin repeats. Cell.

[53] Weigel RR, Bauscher C, Pfitzner AJ, Pfitzner UM (2001). NIMIN-1, NIMIN-2 and NIMIN-3, members of a novel family of proteins from Arabidopsis that interact with NPR1/NIM1, a key regulator of systemic acquired resistance in plants. Plant Mol Biol.

[54] Asai T, Tena G, Plotnikova J, Willmann MR, Chiu WL, Gomez-Gomez L (2002). MAP kinase signalling cascade in Arabidopsis innate immunity. Nature.

[55] Yi SY, Shirasu K, Moon JS, Lee SG, Kwon SY (2014). The activated SA and JA signaling pathways have an influence on flg22-triggered oxidative burst and callose deposition. PLoS One.

[56] Volz R, Kim SK, Mi J, Mariappan KG, Guo X, Bigeard J (2018). The Trihelix transcription factor GT2-like 1 (GTL1) promotes salicylic acid metabolism, and regulates bacterial-triggered immunity. PLoS Genet.

[57] Pre M, Atallah M, Champion A, De Vos M, Pieterse CM, Memelink J (2008). The AP2/ERF domain transcription factor ORA59 integrates jasmonic acid and ethylene signals in plant defense. Plant Physiol.

[58] Berrocal-Lobo M, Molina A, Solano R (2002). Constitutive expression of ETHYLENE-RESPONSE-FACTOR1 in Arabidopsis confers resistance to several necrotrophic fungi. Plant J.

[59] Penninckx IA, Thomma BP, Buchala A, Metraux JP, Broekaert WF (1998). Concomitant activation of jasmonate and ethylene response pathways is required for induction of a plant defensin gene in Arabidopsis. Plant Cell.

[60] Lim PO, Kim HJ, Nam HG (2007). Leaf senescence. Annu Rev Plant Biol.

[61] Dhar N, Caruana J, Erdem I, Subbarao KV, Klosterman SJ, Raina R (2020). The Arabidopsis SENESCENCE-ASSOCIATED GENE 13 regulates dark-induced senescence and plays contrasting roles in defense against bacterial and fungal pathogens. Mol Plant Microbe Interact.

[62] Chen LQ, Lin IW, Qu XQ, Sosso D, McFarlane HE, Londono A (2015). A cascade of sequentially expressed sucrose transporters in the seed coat and endosperm provides nutrition for the Arabidopsis embryo. Plant Cell.

[63] Weaver LM, Gan S, Quirino B, Amasino RM (1998). A comparison of the expression patterns of several senescence-associated genes in response to stress and hormone treatment. Plant Mol Biol.

[64] Callard D, Axelos M, Mazzolini L (1996). Novel molecular markers for late phases of the growth cycle of Arabidopsis thaliana cell-suspension cultures are expressed during organ senescence. Plant Physiol.

[65] Queval G, Thominet D, Vanacker H, Miginiac-Maslow M, Gakiere B, Noctor G (2009). H2O2-activated up-regulation of glutathione in Arabidopsis involves induction of genes encoding enzymes involved in cysteine synthesis in the chloroplast. Mol Plant.

[66] Rahantaniaina MS, Tuzet A, Mhamdi A, Noctor G (2013). Missing links in understanding redox signaling via thiol/disulfide modulation: how is glutathione oxidized in plants?. Front Plant Sci.

[67] Sewelam N, Kazan K, Hüdig M, Maurino VG, Schenk PM. The AtHSP17.4C1 gene expression is mediated by diverse signals that link biotic and abiotic stress factors with ROS and can be a useful molecular marker for oxidative stress. Int J Mol Sci. 2019;20:3201. 10.3390/ijms20133201 PMC6650836 31261879

[68] Rentel MC, Lecourieux D, Ouaked F, Usher SL, Petersen L, Okamoto H (2004). OXI1 kinase is necessary for oxidative burst-mediated signalling in Arabidopsis. Nature.

[69] Zhou X, Jiang Y, Yu D (2011). WRKY22 transcription factor mediates dark-induced leaf senescence in Arabidopsis. Mol Cells.

[70] Miao Y, Laun T, Zimmermann P, Zentgraf U (2004). Targets of the WRKY53 transcription factor and its role during leaf senescence in Arabidopsis. Plant Mol Biol.

[71] Jia L, Xu W, Li W, Ye N, Liu R, Shi L (2013). Class III peroxidases are activated in proanthocyanidin-deficient Arabidopsis thaliana seeds. Ann Bot.

[72] Apel K, Hirt H (2004). Reactive oxygen species: metabolism, oxidative stress, and signal transduction. Annu Rev Plant Biol.

[73] Somssich IE, Wernert P, Kiedrowski S, Hahlbrock K (1996). Arabidopsis thaliana defense-related protein ELI3 is an aromatic alcohol:NADP+ oxidoreductase. Proc Natl Acad Sci U S A.

[74] Yamauchi Y, Hasegawa A, Taninaka A, Mizutani M, Sugimoto Y (2011). NADPH-dependent reductases involved in the detoxification of reactive carbonyls in plants. J Biol Chem.

[75] Noctor G, Foyer CH (1998). ASCORBATE AND GLUTATHIONE: keeping active oxygen under control. Annu Rev Plant Physiol Plant Mol Biol.

[76] Mhamdi A, Noctor G, Baker A (2012). Plant catalases: peroxisomal redox guardians. Arch Biochem Biophys.

[77] Pilon M, Ravet K, Tapken W (2011). The biogenesis and physiological function of chloroplast superoxide dismutases. Biochim Biophys Acta.

[78] Alscher RG, Erturk N, Heath LS (2002). Role of superoxide dismutases (SODs) in controlling oxidative stress in plants. J Exp Bot.

[79] Nounjan N, Nghia PT, Theerakulpisut P (2012). Exogenous proline and trehalose promote recovery of rice seedlings from salt-stress and differentially modulate antioxidant enzymes and expression of related genes. J Plant Physiol.

[80] Torres MA, Dangl JL (2005). Functions of the respiratory burst oxidase in biotic interactions, abiotic stress and development. Curr Opin Plant Biol.

[81] Torres MA, Dangl JL, Jones JD (2002). Arabidopsis gp91phox homologues AtrbohD and AtrbohF are required for accumulation of reactive oxygen intermediates in the plant defense response. Proc Natl Acad Sci U S A.

[82] Beaugelin I, Chevalier A, D'Alessandro S, Ksas B, Novak O, Strnad M (2019). OXI1 and DAD regulate light-induced cell death antagonistically through Jasmonate and salicylate levels. Plant Physiol.

[83] Černý M, Habánová H, Berka M, Luklová M, Brzobohatý B. Hydrogen Peroxide: Its Role in Plant Biology and Crosstalk with Signalling Networks. Int J Mol Sci. 2018;19:2812. 10.3390/ijms19092812 PMC6163176 30231521

[84] Szklarczyk D, Franceschini A, Wyder S, Forslund K, Heller D, Huerta-Cepas J (2015). STRING v10: protein-protein interaction networks, integrated over the tree of life. Nucleic Acids Res.

[85] Galletti R, Ferrari S, De Lorenzo G (2011). Arabidopsis MPK3 and MPK6 play different roles in basal and oligogalacturonide- or flagellin-induced resistance against Botrytis cinerea. Plant Physiol.

[86] Su J, Yang L, Zhu Q, Wu H, He Y, Liu Y (2018). Active photosynthetic inhibition mediated by MPK3/MPK6 is critical to effector-triggered immunity. PLoS Biol.

[87] Meents AK, Mithöfer A. Plant–Plant Communication: Is There a Role for Volatile Damage-Associated Molecular Patterns? Front Plant Sci. 2020;11:583275. https://doi.org/10.3389/fpls.2020.583275. 10.3389/fpls.2020.583275 PMC7593327 33178248

[88] Volz R, Kim SK, Mi J, Mariappan KG, Siodmak A, Al-Babili S, et al. A Chimeric IDD4 Repressor Constitutively Induces Immunity in Arabidopsis via the Modulation of Salicylic Acid and Jasmonic Acid Homeostasis. Plant Cell Physiol. 2019;60:1536–55. 10.1093/pcp/pcz057 30989238

[89] Liu Y, Ren D, Pike S, Pallardy S, Gassmann W, Zhang S (2007). Chloroplast-generated reactive oxygen species are involved in hypersensitive response-like cell death mediated by a mitogen-activated protein kinase cascade. Plant J.

[90] Smith JM, Heese A (2014). Rapid bioassay to measure early reactive oxygen species production in Arabidopsis leave tissue in response to living pseudomonas syringae. Plant Methods.

