# Supplementary figures and images for: Lyso-phosphatidylethanolamine primes the plant immune system and promotes basal resistance against hemibiotrophic pathogens
Source: BMC Biotechnol. 2021 Feb 3;21:12. doi: 10.1186/s12896-020-00661-8 (PMC7856808; doi:10.1186/s12896-020-00661-8)

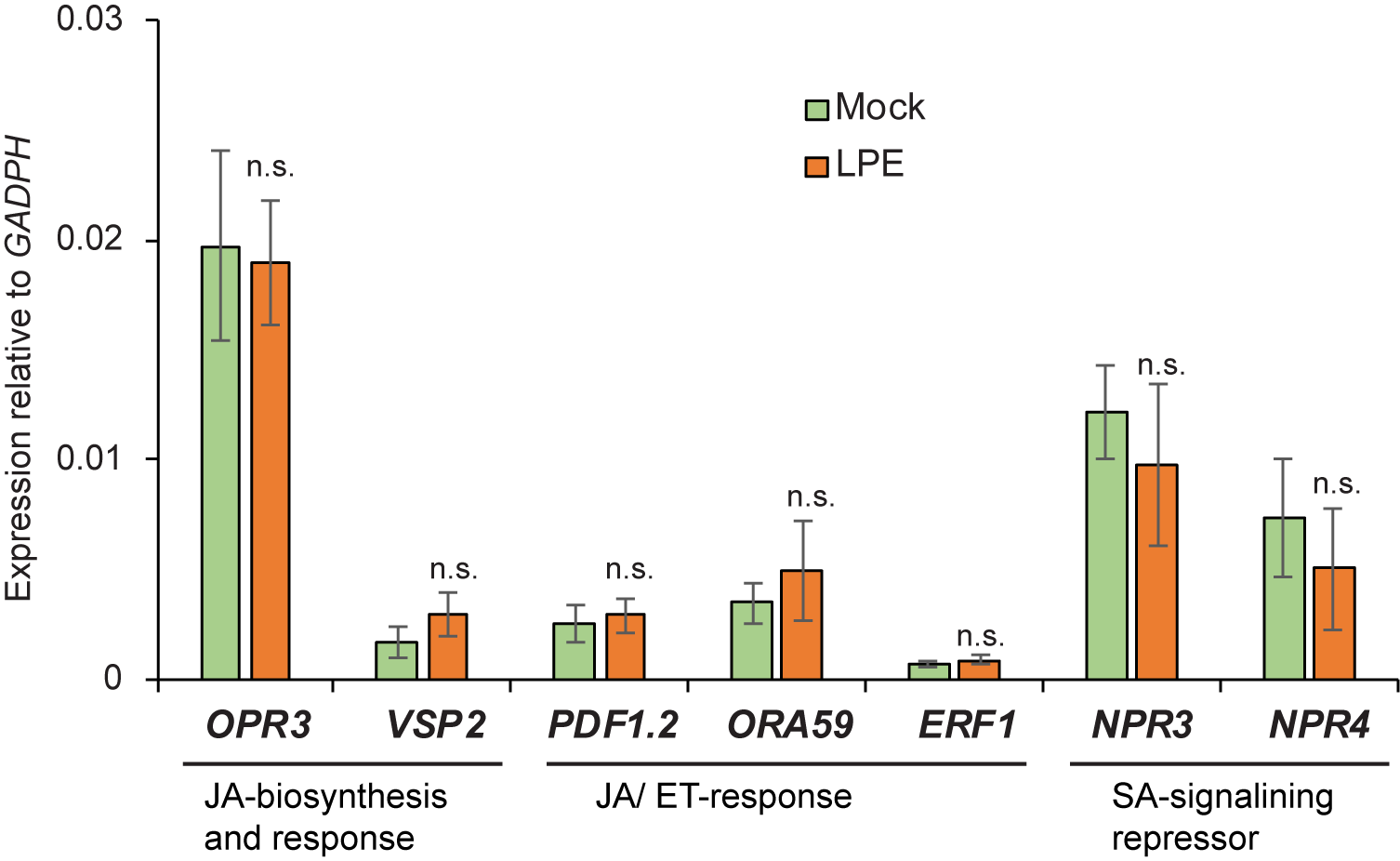

Supplement: Supplementary file 1 — Additional file 1: Figure S1. Genes, involved in JA/ET metabolism and SA signalling repression, are not differentially expressed after LPE-treatment. [file 12896_2020_661_MOESM1_ESM.tif]
